# Supplementary material for: Metasurface-empowered snapshot hyperspectral imaging with convex/deep (CODE) small-data learning theory
Source: Nat Commun. 2023 Nov 1;14:6979. doi: 10.1038/s41467-023-42381-5 (PMC10620425; doi:10.1038/s41467-023-42381-5)
Supplement: Supplementary file 1 — Supplementary Information [file 41467_2023_42381_MOESM1_ESM.pdf]

Supplementary Materials

# Metasurface-empowered Snapshot Hyperspectral Imaging with Convex/Deep (CODE) Small-Data Learning Theory

Chia-Hsiang Lin[1,2,†*], Shih-Hsiu Huang[3†], Ting-Hsuan Lin[1], and Pin Chieh Wu[3,4*]

[1]Department of Electrical Engineering, National Cheng Kung University, Tainan 70101, Taiwan

[2]Miin Wu School of Computing, National Cheng Kung University, Tainan 70101, Taiwan

[3]Department of Photonics, National Cheng Kung University, Tainan 70101, Taiwan

[4]Center for Quantum Frontiers of Research & Technology (QFort), National Cheng Kung University, Tainan 70101, Taiwan

[†]These authors contribute equally to this work

[*]E-mail address: chiahsiang.steven.lin@gmail.com; pcwu@gs.ncku.edu.tw

**Supplementary Table 1. Parameters of the DBR substrate.** The first layer is attached to a glass substrate.

| Layer | Material | Thickness (nm) |
|---|---|---|
| 1 | $Ta_2O_5$ | 109.99 |
| 2 | $SiO_2$ | 124.77 |
| 3 | $Ta_2O_5$ | 107.14 |
| 4 | $SiO_2$ | 158.56 |
| 5 | $Ta_2O_5$ | 102.9 |
| 6 | $SiO_2$ | 155.54 |
| 7 | $Ta_2O_5$ | 98.34 |
| 8 | $SiO_2$ | 159.03 |
| 9 | $Ta_2O_5$ | 92.74 |
| 10 | $SiO_2$ | 155.72 |
| 11 | $Ta_2O_5$ | 92.95 |
| 12 | $SiO_2$ | 153.23 |
| 13 | $Ta_2O_5$ | 92.95 |
| 14 | $SiO_2$ | 154.03 |
| 15 | $Ta_2O_5$ | 87.94 |
| 16 | $SiO_2$ | 147 |
| 17 | $Ta_2O_5$ | 83.66 |
| 18 | $SiO_2$ | 143.64 |
| 19 | $Ta_2O_5$ | 96.32 |
| 20 | $SiO_2$ | 148.54 |
| 21 | $Ta_2O_5$ | 91.84 |
| 22 | $SiO_2$ | 133.61 |
| 23 | $Ta_2O_5$ | 92.66 |
| 24 | $SiO_2$ | 136.96 |
| 25 | $Ta_2O_5$ | 84.61 |
| 26 | $SiO_2$ | 148.64 |
| 27 | $Ta_2O_5$ | 83.37 |
| 28 | $SiO_2$ | 138.47 |
| 29 | $Ta_2O_5$ | 83.98 |
| 30 | $SiO_2$ | 129.7 |
| 31 | $Ta_2O_5$ | 76.24 |
| 32 | $SiO_2$ | 124.29 |
| 33 | $Ta_2O_5$ | 78.88 |
| 34 | $SiO_2$ | 124.36 |
| 35 | $Ta_2O_5$ | 85.68 |
| 36 | $SiO_2$ | 104.61 |
| 37 | $Ta_2O_5$ | 76.02 |
| 38 | $SiO_2$ | 115.39 |
| 39 | $Ta_2O_5$ | 60.64 |
| 40 | $SiO_2$ | 140.42 |
| 41 | $Ta_2O_5$ | 71.45 |
| 42 | $SiO_2$ | 122.02 |
| 43 | $Ta_2O_5$ | 70.6 |
| 44 | $SiO_2$ | 101.12 |
| 45 | $Ta_2O_5$ | 75.26 |
| 46 | $SiO_2$ | 109.09 |
| 47 | $Ta_2O_5$ | 62.35 |
| 48 | $SiO_2$ | 121.3 |
| 49 | $Ta_2O_5$ | 76.04 |
| 50 | $SiO_2$ | 93.95 |
| 51 | $Ta_2O_5$ | 70.16 |
| 52 | $SiO_2$ | 105.66 |
| 53 | $Ta_2O_5$ | 58 |
| 54 | $SiO_2$ | 90.66 |
| 55 | $Ta_2O_5$ | 57.38 |
| 56 | $SiO_2$ | 112.04 |
| 57 | $Ta_2O_5$ | 58.72 |
| 58 | $SiO_2$ | 92.42 |
| 59 | $Ta_2O_5$ | 53.15 |
| 60 | $SiO_2$ | 97.33 |
| 61 | $Ta_2O_5$ | 63.75 |
| 62 | $SiO_2$ | 97.5 |
| 63 | $Ta_2O_5$ | 52.52 |
| 64 | $SiO_2$ | 83.01 |
| 65 | $Ta_2O_5$ | 53.42 |
| 66 | $SiO_2$ | 114.97 |
| 67 | $Ta_2O_5$ | 58.74 |
| 68 | $SiO_2$ | 85 |
| 69 | $Ta_2O_5$ | 47.55 |
| 70 | $SiO_2$ | 76.17 |
| 71 | $Ta_2O_5$ | 52.92 |
| 72 | $SiO_2$ | 75.76 |
| 73 | $Ta_2O_5$ | 46.98 |
| 74 | $SiO_2$ | 72.6 |
| 75 | $Ta_2O_5$ | 52.67 |
| 76 | $SiO_2$ | 73.85 |
| 77 | $Ta_2O_5$ | 48.53 |
| 78 | $SiO_2$ | 71.72 |
| 79 | $Ta_2O_5$ | 45.47 |
| 80 | $SiO_2$ | 73.33 |
| 81 | $Ta_2O_5$ | 50.24 |
| 82 | $SiO_2$ | 72.06 |
| 83 | $Ta_2O_5$ | 48.33 |
| 84 | $SiO_2$ | 69.58 |
| 85 | $Ta_2O_5$ | 48.78 |
| 86 | $SiO_2$ | 68.76 |
| 87 | $Ta_2O_5$ | 43.83 |

**Supplementary Table 2.** Quantitative comparison among the proposed CODE, the pure CO imaging technique (i.e., CO-TV) and the pure DE imaging techniques (i.e., DE-1 and DE-2), where TV refers to total variation. DE-1 and DE-2 are also known as MST++ and HRNet, respectively. The smaller the value of RMSE and SAM, the better the reconstruction fidelity (boldfaced number indicates the best performance). See Supplementary Note 4 and Supplementary Note 5 for more details.

| Data | Methods | RMSE | SAM | Time (sec.) |
|---|---|---|---|---|
| Potted Flower (Non-filtered) | CODE | **0.01051** | **6.110** | 3.94 |
| | CO-TV | 0.03821 | 49.955 | 163.15 |
| | DE-1 | 0.02334 | 9.819 | **1.01** |
| | DE-2 | 0.02632 | 9.085 | 3.55 |
| Hatchet (Non-filtered) | CODE | **0.00896** | **4.889** | 3.72 |
| | CO-TV | 0.02228 | 50.352 | 171.26 |
| | DE-1 | 0.02046 | 9.166 | **1.35** |
| | DE-2 | 0.02072 | 5.361 | 3.72 |
| Potted Flower (Filtered) | CODE | **0.00898** | **5.831** | 4.04 |
| | CO-TV | 0.03814 | 49.912 | 160.12 |
| | DE-1 | 0.02646 | 11.662 | **1.02** |
| | DE-2 | 0.02661 | 10.227 | 3.91 |
| Hatchet (Filtered) | CODE | **0.00649** | **4.167** | 4.01 |
| | CO-TV | 0.02215 | 50.287 | 166.30 |
| | DE-1 | 0.02157 | 9.675 | **1.37** |
| | DE-2 | 0.01895 | 6.554 | 4.05 |

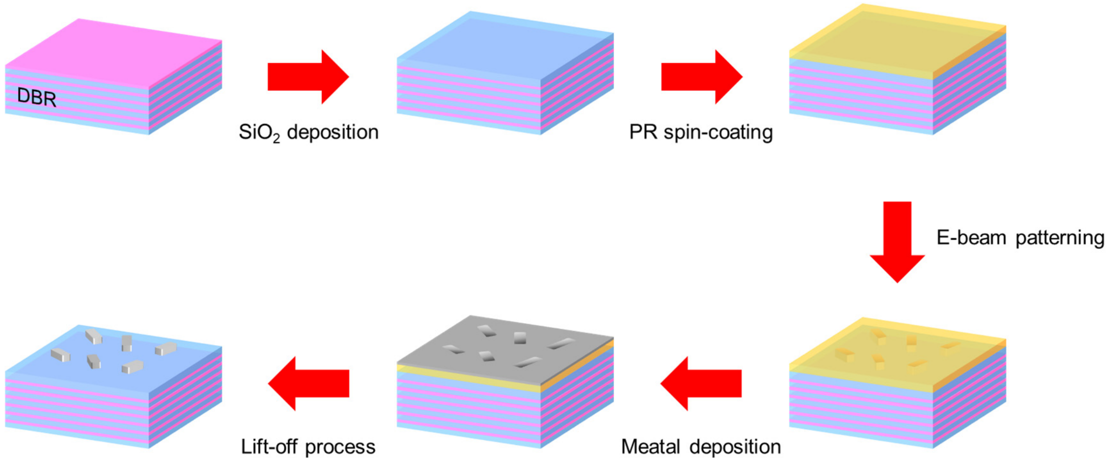


**Supplementary Figure 1.** Fabrication processes of multi-resonant metasurface.

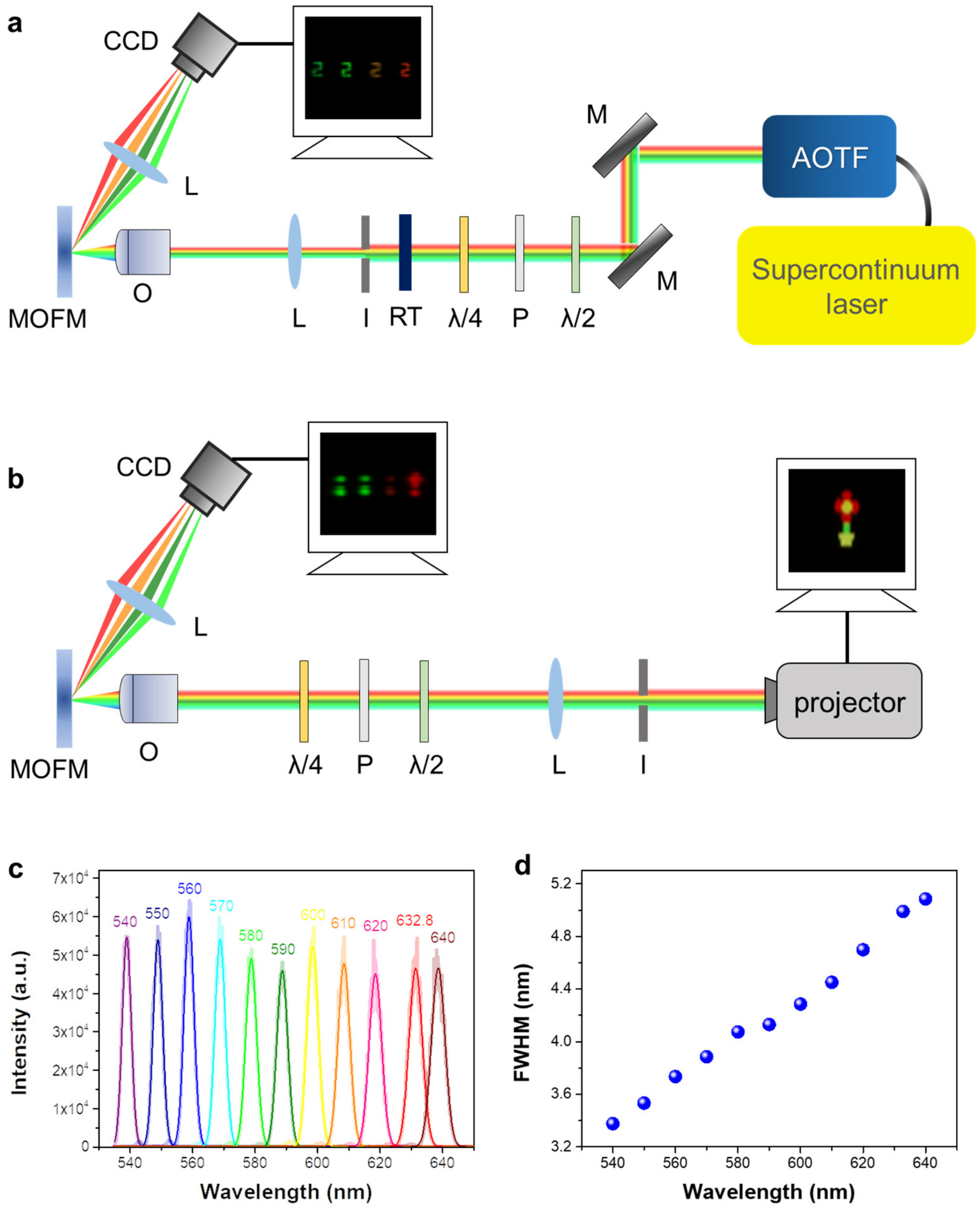


**Supplementary Figure 2. Optical setup for the imaging characterization of multi-wavelength off-axis focusing meta-mirror. a** A supercontinuum laser (NKT Photonics FIU-15) combined with an acousto-optic tunable filter (AOTF, SuperK SELECT) is utilized to select the wavelength in the visible. In this case, resolution targets with different-shaped apertures are used as the object. **b** To demonstrate the snapshot hyperspectral imaging capability of the MOFM, full-color images from a projector are used as the objects. A half-wave plate (Thorlabs AHWP05M-600), a linear polarizer (Thorlabs LPVISE100-A), and a quarter-wave plate (Thorlabs AQWP05M-600) are used to determine the polarization state. M: mirror; I: iris; $\lambda/2$: half-wave plate; P: linear polarizer; λ/4: quarter-wave plate; O: objective (Mitutoyo 10× magnification with 0.28 numerical aperture); RT: resolution targets; L: Lens. **c** The optical spectrum of the laser beams emitted by the AOTF. The wavelength range shown is limited to 530-650 nm due to the capabilities of the used micro-spectrometer (Phekda Series, PD) for measurement. **d** The peak-fitting FWHM of the laser beams carried out from (c).

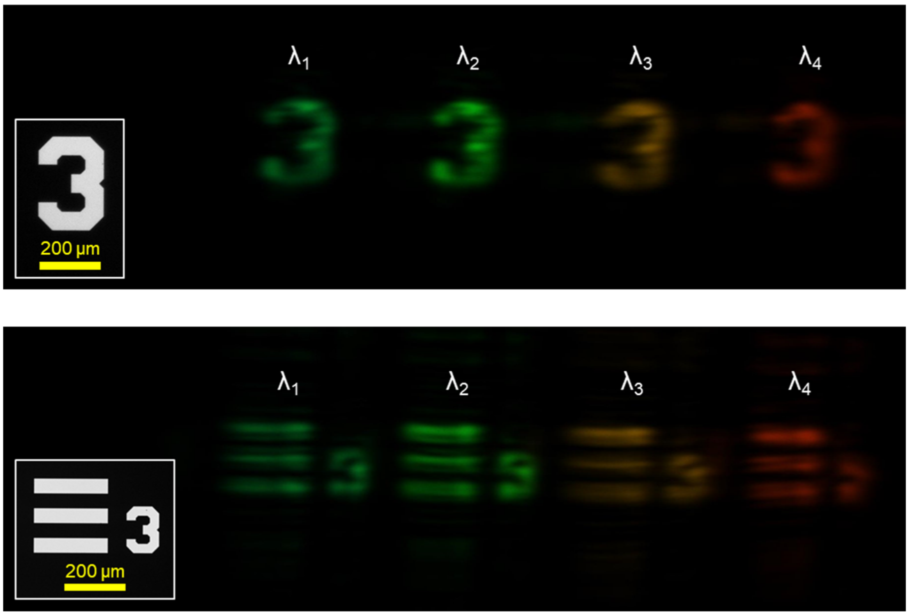


**Supplementary Figure 3. Imaging demonstration of the MOFM using laser source.** Experimentally captured images at 4 wavelength bands. The left panel presents the optical microscopic image of the object. $\lambda_1$ = 513 nm, $\lambda_2$ = 549 nm, $\lambda_3$ = 593 nm, $\lambda_4$ = 633 nm.

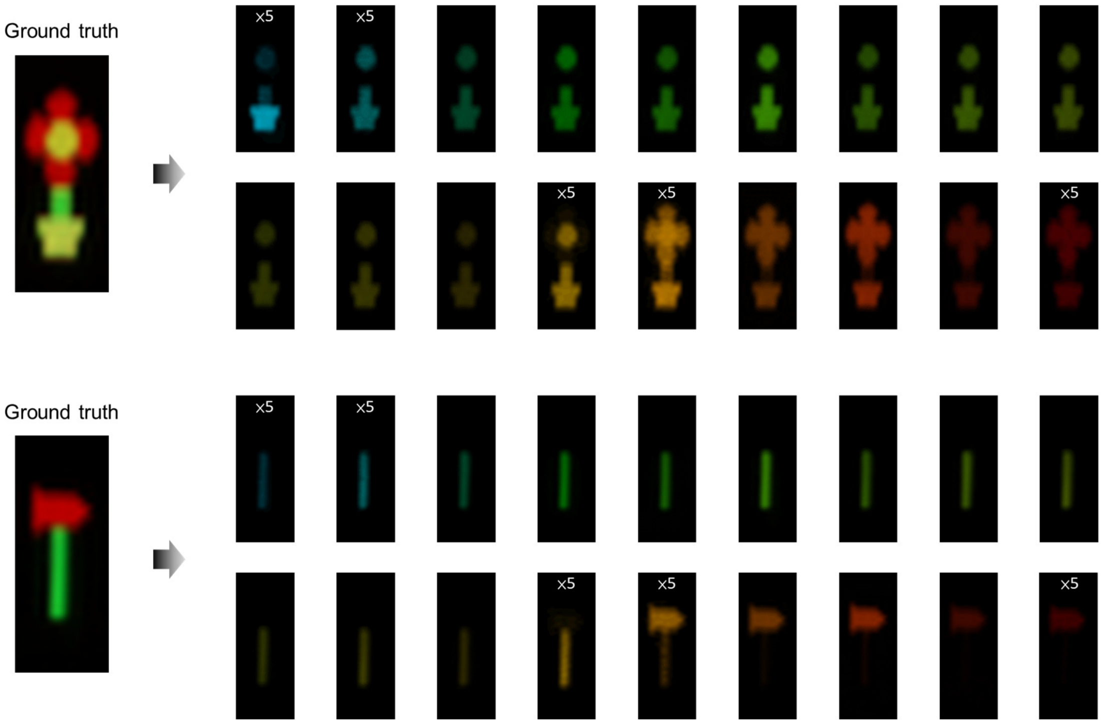


**Supplementary Figure 4. Ground truth of the  hyperspectral images.** The original single wavelength images of two objects. Each ground truth is obtained by using a 10-nm-width band-pass spectral filter. The center wavelengths from left to right and top to bottom are 480, 490, 500, 510, 520, 532, 540, 550, 560, 570, 580, 590, 600, 610, 620, 632.8, 640, 650 nm. Top panel: potted flower. Bottom panel: hatchet.

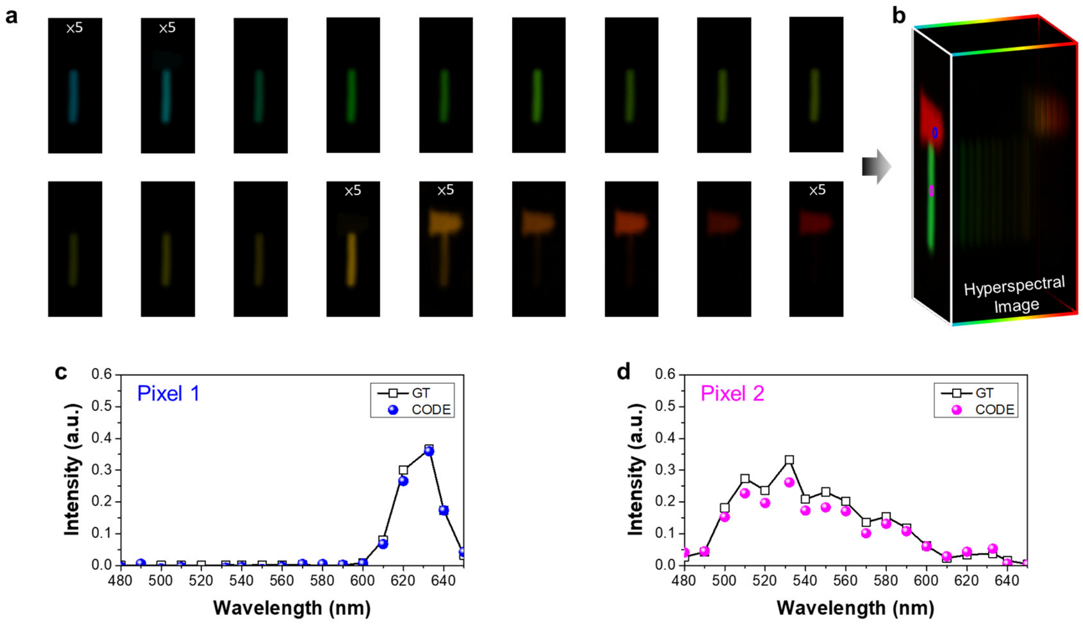


**Supplementary Figure 5. Snapshot hyperspectral imaging for the hatchet. a** Experimentally demonstrated hyperspectral imaging dataset with a single metasurface chip. The center wavelengths are 480, 490, 500, 510, 520, 532, 540, 550, 560, 570, 580, 590, 600, 610, 620, 632.8, 640, 650 nm. **b** Reconstructed image of the hatchet. **c, d** Spectral data at two pixels highlighted in (b). The ground truth (GT) spectral results are provided for comparison.

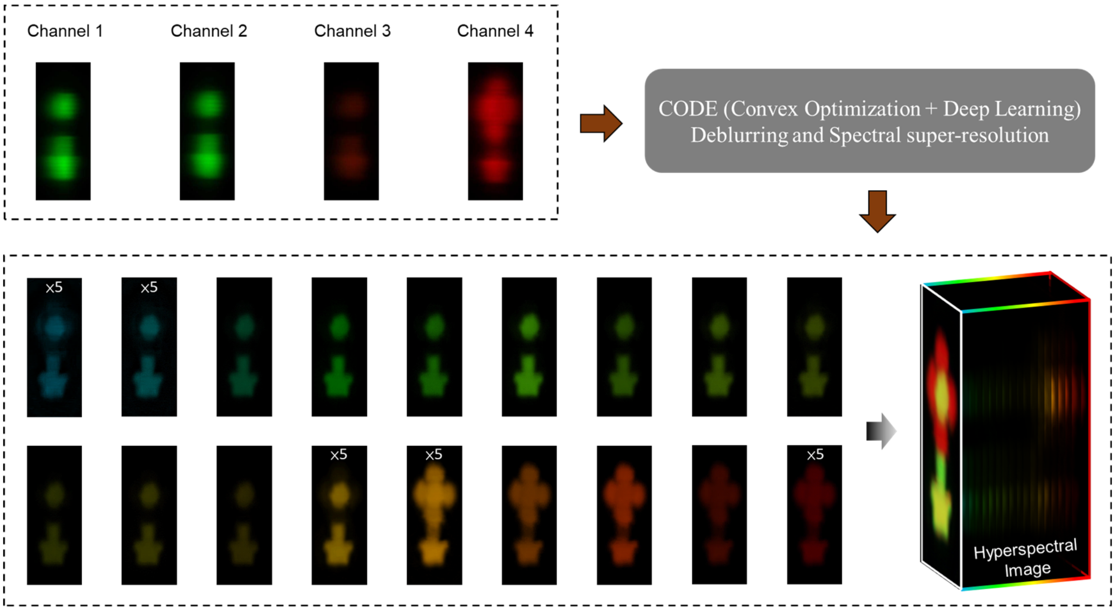


**Supplementary Figure 6. Hyperspectral imaging for the potted flower.** The top-left panel shows the acquired 4-band multispectral image with band pass spectral color filters. The bottom panel shows the computed hyperspectral imaging dataset using the 4-band multispectral image with the CODE small-data learning theory. The center wavelengths of the hyperspectral image dataset are 480, 490, 500, 510, 520, 532, 540, 550, 560, 570, 580, 590, 600, 610, 620, 632.8, 640, 650 nm.

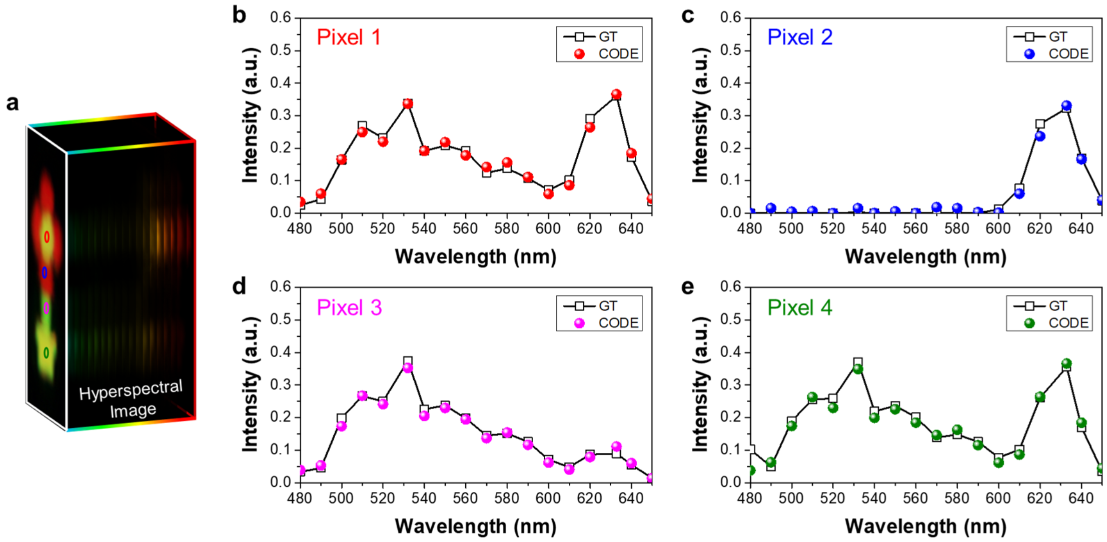


**Supplementary Figure 7. Hyperspectral imaging performance for the potted flower. a** Reconstructed image of the potted flower, which is carried out from Supplementary Fig. 6. **b-e** Spectral data at four pixels highlighted in (a). The ground truth (GT) spectral results are provided for comparison.

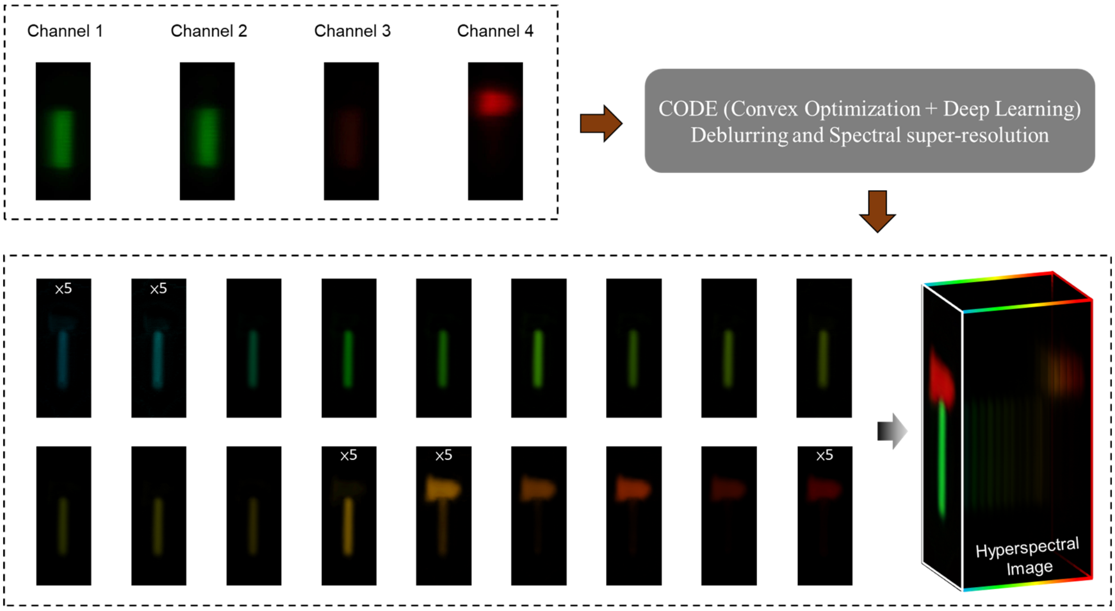


**Supplementary Figure 8. Hyperspectral imaging for the hatchet.** The top-left panel shows the acquired 4-band multispectral image with band pass spectral color filters. The bottom panel shows the computed hyperspectral imaging dataset using the 4-band multispectral image with the CODE small-data learning theory. The center wavelengths of the hyperspectral image dataset are 480, 490, 500, 510, 520, 532, 540, 550, 560, 570, 580, 590, 600, 610, 620, 632.8, 640, 650 nm.

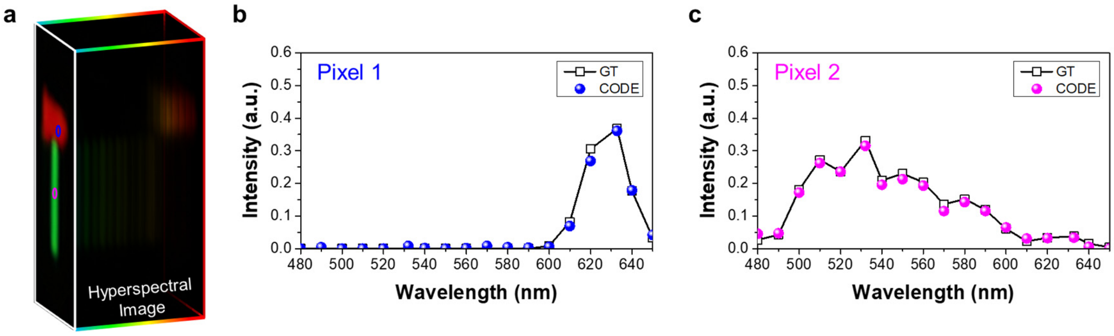


**Supplementary Figure 9. Hyperspectral imaging performance for the hatchet. a** Reconstructed image of the hatchet, which is carried out from Supplementary Fig. 8. **b, c** Spectral data at two pixels highlighted in (a). The ground truth (GT) spectral results are provided for comparison.

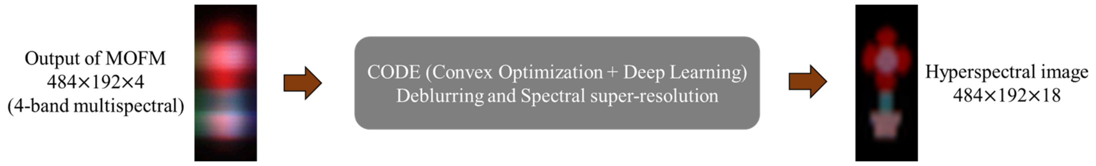


**Supplementary Figure 10. Computational process for the hyperspectral imaging.** The left plot $Y$ is acquired by the proposed MOFM. Our imaging technique applies the CODE learning theory, which blends the advantages of CO and DE, to perform the spectral super-resolution to computationally obtain the deblurred hyperspectral image $X$ (the right plot). The CODE technique is detailed in Fig. 4 in the main article.

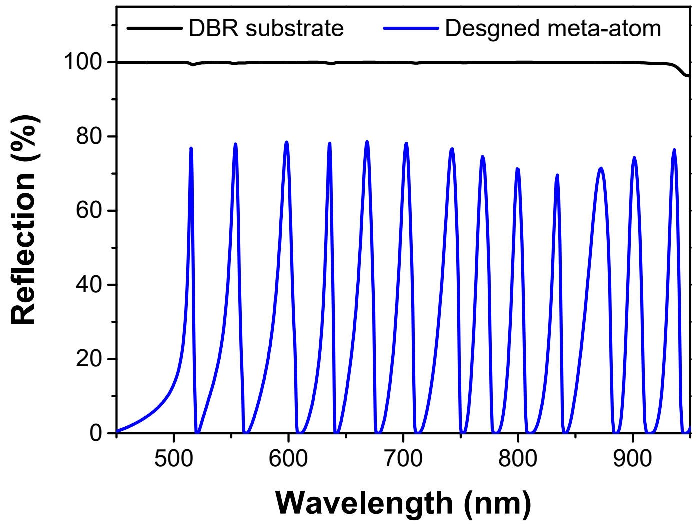


**Supplementary Figure 11. Optical spectrum of the multi-resonant meta-atom and the DBR substrate.** The blue curve shows the numerical LCP-to-RCP reflection spectrum of the designed multi-resonant meta-atom. An Al nano-rod (length = 170 nm, width = 90 nm, thickness = 50 nm, period = 200 nm, thickness of $SiO_2$ spacer = 135 nm) array standing on a DBR substrate is optimized to possess multiple high-Q resonant peaks across the spectral window from 450 nm to 930 nm. The black curve represents the reflection spectrum of the bare DBR substrate.

**Supplementary Note 1: Implementation of transverse chromatic aberration**

In the case of a regular metalens or a focusing meta-mirror, which exhibits longitudinal chromatic aberration, capturing color images in a single-shot measurement is not suitable due to the multispectral images appearing at different focal planes for different wavelength channels (refer to Supplementary Figure 12a). To overcome this limitation, we propose incorporating transverse chromatic aberration into the focusing meta-mirror. To achieve this, a multi-wavelength meta-mirror is specifically designed with an off-axis focusing effect. By introducing transverse chromatic aberration, we can enable the capture of color images in a single-shot measurement, improving the imaging capabilities of the meta-mirror. Building upon previous research conducted[1], the phase distribution of an off-axis meta-mirror/metalens can be simplified as follows:

$$\Phi_{\text{metalens}}\left(r_p, \varphi_p\right) = \frac{2\pi}{\lambda_d}\left(f - \sqrt{f^2 + {r_p}^2 - 2 r_p f \sin\theta_f \cos\varphi_p}\right) \tag{S1}$$

where $\lambda_d$ is the designed central wavelength, $f$ is the focal length for $\lambda_d$, and $\theta_f$ represents polar angle. $r_p$ represents the distance between the meta-mirror center and an arbitrary position on the meta-mirror surface. $\varphi_p$ denotes the angle between the $x$-axis and the line connecting the center of the meta-mirror to the arbitrary position on the meta-mirror surface. In our specific case, the focusing meta-mirror is designed to operate at a central wavelength of 593 nm with a focal length of 7.5 mm. This design enables the formation of color images with different wavelength channels that are away from the central wavelength. As a result, these color images appear on the screen along the dispersion direction, as shown in Supplementary Figure 12b. This characteristic is advantageous for the development of snapshot multispectral imaging, where multiple wavelength channels can be captured simultaneously in a single shot.

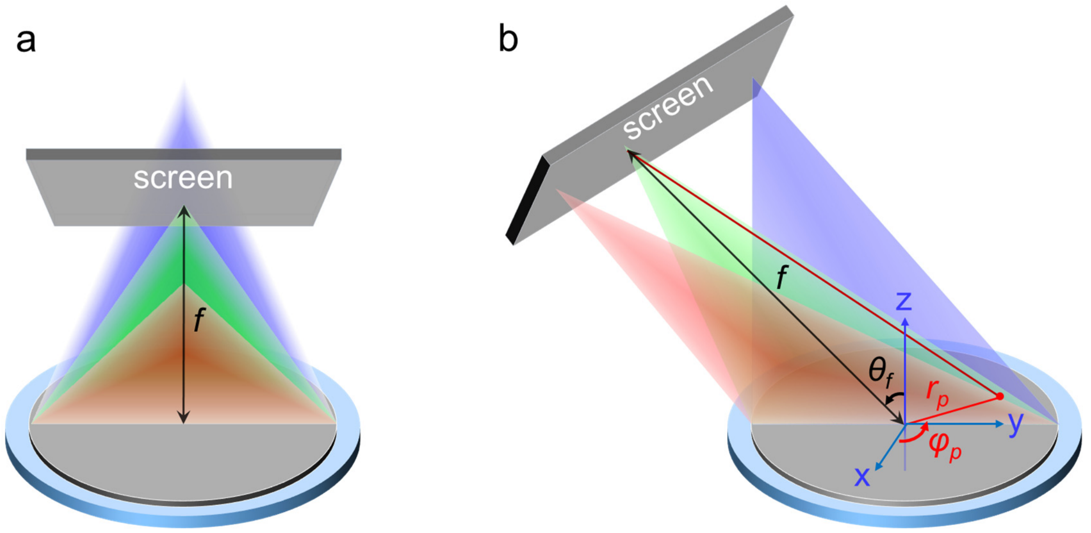


**Supplementary Figure 12. Schematic illustration of the chromatic meta-mirror. a** On-axis dispersive meta-mirror. **b** Off-axis dispersive meta-mirror. The $\theta_f$ is designed at 45° for 593 nm.

**Supplementary Note 2: Impact of the incident angle on the 4-band multispectral imaging**

The incident angle is a critical factor in 4-band multispectral imaging as it affects the reflected angles, lateral separation between color channels, and overall image quality. Supplementary Figure 13 shows additional computed images for various incident angles, clearly demonstrating that larger incident angles lead to increased reflected angles and greater separation between multispectral images. It is notable that at larger incident angles, the images corresponding to longer wavelengths become more blurred due to the presence of strong aberrations. Conversely, for negative incident angles, the images at shorter wavelengths appear blurrier compared to the longer wavelengths that are closer to the central wavelength of the meta-mirror, despite having smaller angles of reflection. These observations emphasize the collective influence of the incident angle, intrinsic characteristics of the meta-mirror, and optical aberrations on the observed blurring effect in multispectral imaging.

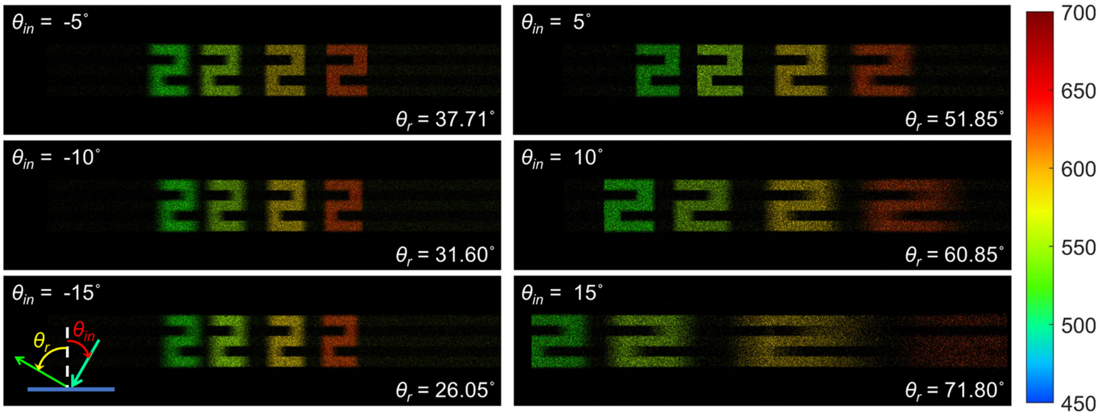


**Supplementary Figure 13. Calculated images of a number "2" at 4 wavelength bands under various angles of incidence.** The $\theta_{\mathrm{in}}$ and $\theta_r$ represent the incident and reflected angles, respectively, for the central wavelength 593 nm.

**Supplementary Note 3: Light sources used for white balance**

Supplementary Figure 14 provides a comparison of the optical spectra emitted by two different light sources used for white balance. The projector shows a lower intensity in the green color range compared to the blue and red colors, causing channel 3 (593 nm) in Fig. 3d to appear more reddish. On the other hand, the white LED exhibits a more consistent intensity across the wavelength range corresponding to green to red colors, resulting in the color at channel 3 in Fig. 3c being closer to its true representation. Importantly, we emphasize that the observed color discrepancy does not compromise the accuracy of the multispectral/hyperspectral imaging results. This is because the spatial distribution of wavelength channels in free space ensures that each captured color image corresponds to the specific wavelength channel at its designated spatial position. Additionally, the small-data learning theory employed for obtaining the hyperspectral imaging data cubes reconstructs individual color images for each wavelength band based on the CIE 1931 color space, guaranteeing accuracy in the results regardless of any color differences observed between the images.

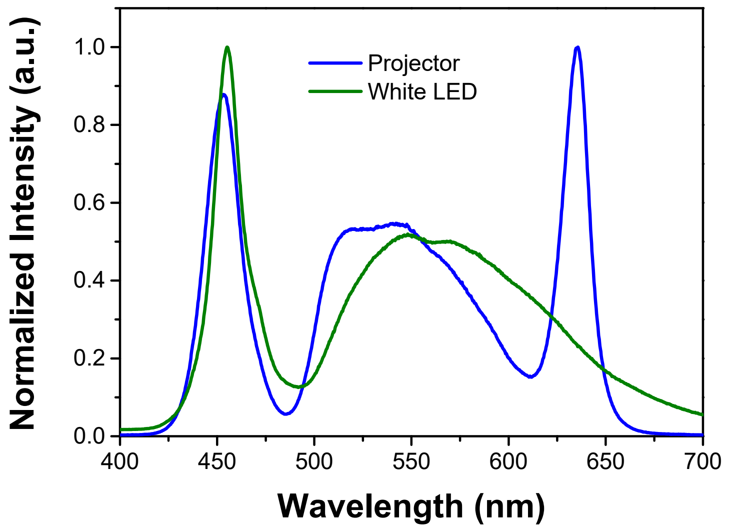


**Supplementary Figure 14. The spectrum of different light sources.** The intensity spectrum of the projector is represented by the blue curve, while the white LED is represented by the olive curve. A white background is used when measuring the optical spectrum of the projector.

**Supplementary Note 4: CODE-based computational imaging**

Let $Y \in \mathbb{R}^{4\times L}$ be the metasurface-acquired $L$-pixel $4$-band multispectral image, from which we aim to reconstruct the corresponding deblurred hyperspectral image $X \in \mathbb{R}^{M\times L}$ (i.e., the target image), where $M$ is the number of hyperspectral bands. Mathematically, $Y$ can be regarded as the blurred low-spectral-resolution counterpart of $X$, and this relation can be concisely modeled as

$$Y = \mathrm{f}_{\mathrm{B}}(DX), \tag{S2}$$

where the spectral response matrix $D$ performs spectral downsampling[2], and $\mathrm{f}_{\mathrm{B}}(\cdot)$ describes the blurring effect caused by the multi-wavelength meta-mirror.

We aim to infer the target image $X$ from the metasurface-acquired information $Y$ computationally. Two commonly seen approaches for solving this imaging inverse problem are convex optimization (CO) and deep learning (DE). However, purely using CO would lead to a math-heavy optimization procedure[3] and thus a slow algorithm, especially under the hardly tractable blurring effect $\mathrm{f}_{\mathrm{B}}$. Also, purely using DE requires the support of big data collection, which is expensive or even unavailable. In our application, collecting big data is not economical because of the need for a bunch of color filters. Therefore, we will employ the very recently proposed CODE machine learning theory[3] not only to avoid heavy math but to achieve small-data learning.

As its name suggested, CODE blends the advantages of CO and DE, thereby avoiding the big data required in DE and avoiding the heavy math often encountered in CO. CODE was originally invented for advanced hyperspectral satellite tasks[3] by introducing the so-called $Q$-quadratic norm $\|\cdot\|_Q$ to bridge CO and DE. For the first time, the CODE learning theory will be employed for hyperspectral metasurface imaging; surprisingly, extremely little data (only 18 images, each containing just 484 ×192 pixels) is sufficient for high-quality hyperspectral imaging.

**CODE Formulation**

This subsection will briefly recall the most critical parts of the CODE theory[3], including the design of the convex data-fitting term and the design of deep $Q$-quadratic regularization, *etc*. As the sophisticated metasurface system leads to a non-convex blurring effect, the original version of CODE is not directly applicable. So, DE will be employed to reformulate the MOFM imaging problem. The corresponding CODE-based imaging problem will be formulated hereinafter.

Due to the complicated mechanism of the metasurface system, it is difficult to describe the blurring effect $\mathrm{f}_{\mathrm{B}}$ using an explicit mathematical function. Even with the function $f_B$, the naive data-fitting term $\|Y - \mathrm{f}_{\mathrm{B}}(DX)\|_F$ is non-convex and hard to be efficiently addressed, where $\|\cdot\|_F$ is the Frobenius norm. Thus, we propose to learn the inverse blurring procedure $\mathrm{f}_{\mathrm{B}}^{-1}(\cdot)$ using the deep Transformer model, as will be detailed in **CODE Implementation**. This provides the quantity $\widetilde{Y} \triangleq \mathrm{f}_{\mathrm{B}}^{-1}(Y)$, which can then be used to design a neat convex data-fitting term, i.e., $\|\widetilde{Y} - DX\|_F$, leading to the following CODE-based formulation:

$$X^{\star} = \arg\min_X \ \|\widetilde{Y} - DX\|_F^2 + \mathrm{REG}(X), \tag{S3}$$

where the convex regularization term will be designed later.

Recovering $X$ from $Y$ (or $\widetilde{Y}$) is an ill-posed problem; the regularization term $\mathrm{REG}(X)$ is needed to encourage a physically interpretable solution $X$. To achieve effective regularization, a mathematically sophisticated function is often needed. For example, the challenging super-resolution imaging task for the Sentinel-2 satellite requires a graph-embedded regularization function to encourage a self-similarity solution[4]. In CODE small-data learning[3], the deep $Q$-quadratic regularization function $\|\cdot\|_Q$, with regularization strength controlled by $\lambda > 0$, is employed to design the convex regularization scheme

$$\mathrm{REG}(X) \triangleq \lambda \|X - X_{\mathrm{DE}}\|_Q^2, \tag{S4}$$

as illustrated below. First, $\|\cdot\|_Q$ is a convex function defined as $\|v\|_Q^2 \triangleq v^T Q v$ with $Q$ being some properly designed positive semidefinite (PSD) matrix[5]. Second, as demonstrated in Ref. 3, CODE can use the deep regularizer to extract useful features embedded in the small data; specifically, even if small data can yield a weak DE solution $X_{\mathrm{DE}} \in \mathbb{R}^{M \times L}$ (viewed as a rough imaging result), the CODE theory believes that the weak solution still contains meaningful texture information, and adopts the regularizer $\|\cdot\|_Q$ to extract the information to be injected into the final target image $X$. Overall, $X_{\mathrm{DE}}$ can be viewed as a spectrally upsampled version of $\widetilde{Y}$, and can be obtained just using a simple deep learning network as a rough solution $X_{\mathrm{DE}}$ is sufficient for CODE to yield a good learning result[3]. This simple deep network for mapping $\widetilde{Y}$ to $X_{\mathrm{DE}}$ will be detailed in **CODE Implementation**. As a side remark, though CODE has achieved numerous challenging hyperspectral signal processing tasks in very recent literature, including the tensor completion (i.e., image inpainting)[3] and satellite change detection[12], the naïve version of CODE is far from being applicable to the hardly tractable hyperspectral metasurface imaging, for which we further introduced the deep data-fitting (besides the deep regularization in the naïve CODE); this can be graphically illustrated using the notation system built above, as displayed in the Supplementary Figure 15.

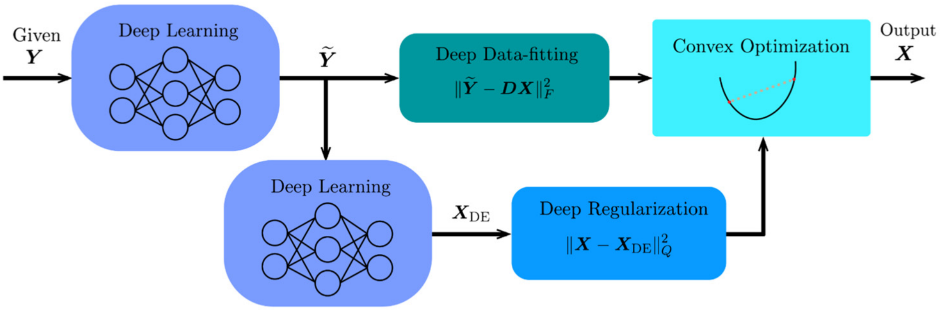


(a) Newly designed CODE for hyperspectral metasurface imaging.

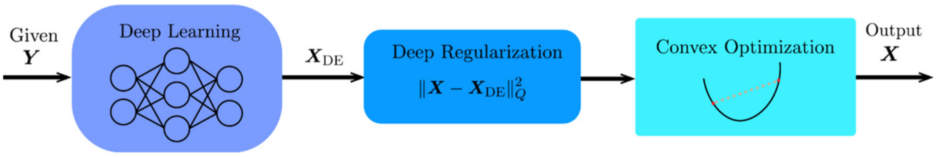


(b) Naïve CODE proposed in Ref. 3.

**Supplementary Figure 15.** The naïve CODE (bottom panel) involves only deep $Q$-norm regularization. To address the hardly tractable hyperspectral metasurface imaging, another Transformer-based deep data-fitting term has been further introduced into the newly designed CODE (top panel).

## CODE Implementation

In this subsection, we need to explain the following implementation details:

- How to design the deep network for learning the inverse blurring procedure $f_B^{-1}(\cdot)$ using the deep Transformer model? How to design the deep network for mapping $\widetilde{Y}$ to $X_{DE}$?
- How to train deep networks using small data? Note that the original CODE theory achieves advanced hyperspectral satellite mission using hundred-scale small data, while we now have just 18 data points; that said, besides CODE, other tricks are needed for effective small-data learning.
- How to design $Q$ and accordingly derive the algorithm with closed-form solutions for the CODE formulation?

The three questions are respectively answered below.

First, considering that the blurring effect caused by the MOFM is intractable and expected to be highly non-linear, we learn the inverse procedure $f_B^{-1}$ using the advanced Transformer technique. The proposed Transformer is deployed using the U-Net structure, as detailed in Figure 4 in the main article, where each Transformer block (T-Block) is also depicted. The T-block revises the Restormer[6] to have better interaction among the feature maps. This upgrades the QKV attention effectiveness gained by interchanging the ordering of the depthwise convolution (Dconv) and the typical convolution block. After the last T-Block that focuses more on spectral attention, we further enhance the spatial features by the novel technique called spatial-spectral domain learning (SDL) module[7], whose output is the desired deblurred multispectral image $\widetilde{Y} = f_B^{-1}(Y)$. Next, we explain how to map the multispectral image $\widetilde{Y}$ to the hyperspectral image $X_{DE}$. As previously discussed, the CODE learning theory does

not require big data and can accept a roughly estimated solution $X_{\mathrm{DE}}$ obtained from small data. For this reason, a simple two-branch convolution neural network (CNN) deployed like Fig. 4 in the main article is sufficient to obtain $X_{\mathrm{DE}}$ for effectively supporting the subsequent deep regularization to be implemented by **Algorithm 1**.

Second, we explain how to achieve effective small-data learning with the standard root mean squared error (RMSE) loss function. The CODE theory works well for hundred-scale data[3], but we now have only 18 data points. So, we invent a new data augmentation technique to support the CODE learning. We first recall that the most commonly seen data augmentation would be based on the image rotation transform (RT). However, RT mainly enhances spatial features and is outside our application. In our application, the most challenging part lies in increasing the number of spectral bands; specifically, the output of the meta-mirror has just 4 bands, and we aim to upsample it to the 18-band hyperspectral image. Based on this observation, we propose to use the color transform (CT), which better captures the problem's nature compared to the RT. Mathematically, given the available small dataset $\mathcal{D} = \{Y_1, Y_2 \ldots, Y_{20}\}$, we can augment it to $\mathcal{D}_{\text{aug}} = \{Y_1, Y_1', Y_2, Y_2', \ldots, Y_{20}, Y_{20}'\}$ in which $Y_i'$ is just a row-shifted version of $Y_i$. Note that in this paper, the rows of the image $Y$ correspond to spectral bands (colors), and columns correspond to pixels; thus, row shift exactly implements the desired CT data augmentation. Our dataset contains 20 pairs of images, each composed of a blurred 4-band multispectral image and a clean 18-band hyperspectral image, both having a spatial size of $484 \times 192$. The 18-band hyperspectral images' spectral wavelengths are 480, 490, 500, 510, 520, 532, 540, 550, 560, 570, 580, 590, 600, 610, 620, 632.8, 640, 650 nm. As discussed above, we use CT to augment the dataset to 40 pairs in $\mathcal{D}_{\text{aug}}$, of which 36 are for training, 2 for validation, and 2 for testing.

**Algorithm 1** The CODE-based Metasurface Hyperspectral Imaging Algorithm

**Given** $\lambda := 0.1$, $\mu := 10$. Initialize $\boldsymbol{s}^0 := \boldsymbol{0}$, $\boldsymbol{d}^0 := \boldsymbol{0}$, and $q := 0$.
**repeat**
  Update $\boldsymbol{z}^{q+1} \triangleq \arg\min_{\boldsymbol{z}} \mathcal{L}(\boldsymbol{s}^q, \boldsymbol{z}, \boldsymbol{d}^q) := \frac{\lambda}{\lambda+\mu}\boldsymbol{s}_{\mathrm{DE}} + \frac{\mu}{\lambda+\mu}(\boldsymbol{s}^q - \boldsymbol{d}^q)$;
  Update $\boldsymbol{s}^{q+1} \triangleq \arg\min_{\boldsymbol{s}} \mathcal{L}(\boldsymbol{s}, \boldsymbol{z}^{q+1}, \boldsymbol{d}^q) := \boldsymbol{I}_L \otimes \boldsymbol{\Psi}^{-1}((\boldsymbol{I}_L \otimes \boldsymbol{E}^T\boldsymbol{D}^T)\widetilde{\boldsymbol{y}} + \mu(\boldsymbol{z}^{q+1} + \boldsymbol{d}^q))$;
  Update $\boldsymbol{d}^{q+1} := \boldsymbol{d}^q + \boldsymbol{z}^{q+1} - \boldsymbol{s}^{q+1}$;
  $q := q + 1$;
**until** the predefined stopping criterion is met.
Compute $\boldsymbol{s}^\star := \boldsymbol{s}^q$ and $\boldsymbol{S}^\star := \mathrm{vec}^{-1}_{N\times L}(\boldsymbol{s}^\star)$.
**Output** the imaging result $\boldsymbol{X}^\star := \boldsymbol{E}\boldsymbol{S}^\star$.

Third, we propose a fast imaging algorithm (i.e., **Algorithm 1**) to implement the formulation Eq. (S3) with all the algorithmic steps solved by closed-form solutions. To this end, we first explain how to design the PSD matrix $Q$. It is known that pixels of a hyperspectral image often concentrate on a hyperspectral simplex (i.e., convex hull of the affinely independent hyperspectral signatures of the underlying materials in a given image). This hyperspectral geometry then implies that those hyperspectral pixels often distributed in a low-dimensional hyperspectral subspace[8], motivating us to design $Q$ using the basis vectors of the subspace. Let $E \in \mathbb{R}^{M\times N}$ be an orthogonal basis of the hyperspectral subspace, which can be obtained by applying principal components analysis to the roughly estimated imaging result$X_{\mathrm{DE}}$. Then, we have $X_{\mathrm{DE}} = ES_{\mathrm{DE}}$ for

some eigen-image[4] $S_{\mathrm{DE}} \in \mathbb{R}^{N\times L}$, whose number of parameters is $NL$, which is much fewer than that of the target image $X = ES \in \mathbb{R}^{M\times L}$ in Eq. (S3) and leads to a faster algorithm. Therefore, a trickily designed geometry-driven PSD matrix $Q$ allows us to reformulate the $Q$-norm into the commonly seen (and more easily tractable) L2 norm. To be mathematically rigorous, let $\mathrm{vec}(\cdot)$ be the vectorization operator and $\mathrm{vec}^{-1}(\cdot)$ be the inverse operator, and select the geometry-driven $Q = I_L \otimes (EE^T)$ to build the relation that $\|x - x_{\mathrm{DE}}\|_Q^2$ is proportional to $\|s - s_{\mathrm{DE}}\|_2^2$, which allows us to concisely reformulate Eq. (S3) into the eigenspace as the convex problem:

$$s^\star = \arg\min_s \ \mathrm{DF}(s) + \tfrac{\lambda}{2}\|s - s_{\mathrm{DE}}\|_2^2, \tag{S5}$$

where $\otimes$ is the Kronecker product, $x = \mathrm{vec}(X)$, $x_{\mathrm{DE}} = \mathrm{vec}(X_{\mathrm{DE}})$, $s = \mathrm{vec}(S)$, $s_{\mathrm{DE}} = \mathrm{vec}(S_{\mathrm{DE}})$, and the data-fitting term $\mathrm{DF}(s)$ will be derived below. Starting from the data-fitting term of Eq. (S3), we have

$$\|\widetilde{Y} - DX\|_F^2 = \|\widetilde{Y} - DES\|_F^2 = \|\widetilde{y} - (I_L \otimes DE)s\|_2^2, \tag{S6}$$

where we used the Kronecker product property of $\mathrm{vec}(ABC) = (C^T \otimes A)\mathrm{vec}(B)$ in the second equality. With Eq. (S6), we can further recast the CODE imaging problem into the standard form of alternating direction method of multipliers (ADMM)[5], i.e.,

$$\min_{s=z} \ \tfrac{1}{2}\|\widetilde{y} - (I_L \otimes DE)s\|_2^2 + \tfrac{\lambda}{2}\|z - s_{\mathrm{DE}}\|_2^2. \tag{S7}$$

Then, the ADMM algorithm for solving Eq. (S7) is presented in **Algorithm 1**, where $q$ is the iteration number, $d \in \mathbb{R}^{NL}$ is the newly introduced scaled dual variable, $\Psi \triangleq (E^T D^T DE + \mu I_N)$, and $\mathcal{L}(s,z,d) = \tfrac{1}{2}\|\widetilde{y} - (I_L \otimes DE)s\|_2^2 + \tfrac{\lambda}{2}\|z - s_{\mathrm{DE}}\|_2^2 + \tfrac{\mu}{2}\|s - z - d\|_2^2$ is the augmented Lagrangian of Eq. (S7) with $\mu > 0$ being the penalty parameter. Note that we have derived the closed-form solutions for all the algorithmic steps, as presented in **Algorithm 1**.

We give four remarks to conclude this section:

1. [Remark 1] Although the standard ADMM convergence conditions (see, e.g., Ref. 9) are not straightforwardly seen, the tricky reformulation of Eq. (S7) ensures the full rankness of the linear association between the two primal variables. According to Ref. 5, such a full rankness can directly build the desired convergence guarantee, as theoretically guaranteed as follows:

**Theorem 1** *Our metasurface-empowered hyperspectral imaging algorithm (i.e., Algorithm 1) is guaranteed to converge to an optimal global solution of Eq. (S7).*

2. [Remark 2] Thanks to the closed-form solutions in Lines 3-5 of **Algorithm 1**, the CODE learning theory[3] leads to speedy computational time. Training and inference are executed on the computer facility equipped with a Core-i9-10900K CPU with 3.70-GHz speed, 64-GB random access memory, and NVIDIA GeForce RTX-3090 GPU. The deep learning parts are trained on Python 3.7 with PyTorch 1.10.1, while the convex optimization part is solved

on MATLAB R2021b. With the above facility, including the DE part (about 2.94 seconds) and the CO part (about 0.78 seconds), the overall computational time to complete the metasurface hyperspectral imaging (for a $484 \times 192$ image; cf. Supplementary Figure 10) is just around 3.72 seconds.

3. [Remark 3] One may wonder how such a mathematically simple CODE theory could be so powerful, but by noticing that the $Q$-norm function (used to bridge CO and DE) is actually a more generalized version of the classical Tikhonov regularization function[3], the good performance becomes not so surprising. Note also that the line of blending CO and DE has become a recent trend in solving hardly-tractable challenging imaging inverse problems (e.g., metasurface hyperspectral imaging). Besides our CODE theory, the most remarkable technique in this line is deep plug-and-play (deep PnP)[10, 11]. However, unlike CODE that just needs small data, the deep PnP technique requires big data to implement the deep proximal operator in the convex ADMM algorithm[10, 11]. With this regard, the CODE theory is quite appealing and promising for other challenging imaging applications.

4. [Remark 4] For hardly tractable inverse problems (e.g., restoration of damaged hyperspectral satellite data), merely using either CO or DE is far from being sufficient especially in the absence of big data, as recently demonstrated in the first CODE paper[3]. Merely using DE yields rather weak solution due to the small scale of the available data, but CO (the convex $Q$-norm regularization, in particular) can employ such a weak solution to regularize the final solution thereby obtaining high-quality hyperspectral imaging results. ADMM (resp., ADAM) is not the key and can be replaced by any other suitable CO optimizers (resp., DE optimizers). That's exactly why "ADMM-Adam" has been renamed "CODE" in recent works. For example, the algorithm for hyperspectral satellite change detection (HCD) has been named as CODE-HCD[12], where the detection result obtained by merely using DE was also proven to be rather weak. The value of the CODE framework can indeed be effectively highlighted by comparing the imaging results obtained from CODE with those from other approaches, as can be seen in Supplementary Table 2. We added some new results to illustrate the value of CODE, by showing that directly using only CO or only DE cannot achieve good spectrum recovery imaging. In order to use DE alone, we employ two state-of-the-art spectrum recovery DE techniques, known as MST++ (i.e., DE-1)[13] and HRNet (i.e., DE-2)[14], both of which are trained using exactly the same small data as CODE. To directly use CO only, we need to remove the DE-based $Q$-norm regularization; thus, for this demonstration, we replaced $Q$-norm by the prestigious CO-based total variation (TV) regularization. The resulting algorithm, named CO-TV, is derived and detailed in Supplementary Note 5. The results are summarized in the Supplementary Table 2. One can see that results gained by using only CO or DE individually (i.e., CO-TV,

DE-1 and DE-2) are far from being satisfactory for both the filtered and non-filtered cases. Also, the much more complicated form of TV leads to significantly longer computational time, compared to the $Q$-norm of mathematically simple form.

## Supplementary Note 5: CO-TV computational imaging algorithm

As discussed in Supplementary Note 4, we replaced the DE-based $Q$-norm regularization by the CO-based TV regularization, and the resulting CO-TV algorithm will be derived below. Specifically, the new imaging criterion becomes

$$X^{\star} = \arg\min_X \ \|\widetilde{Y} - DX\|_F^2 + \lambda \mathrm{TV}(X), \tag{S8}$$

where the data-fitting term remains the same, while the regularization term becomes $\mathrm{TV}(X)$ (i.e., the prestigious total variation function)[15]. Then, let us reformulate Eq. (S8) into the standard ADMM-form as

$$\min_{z=x} \ \|\widetilde{y} - (I_L \otimes D)x\|_2^2 + \lambda \mathrm{TV}(z), \tag{S9}$$

where $\otimes$ is the Kronecker product, $x = \mathrm{vec}(X)$, and $\widetilde{y} = \mathrm{vec}(\widetilde{Y})$. We can then solve Eq. (S9) through the ADMM algorithm, as detailed in **Algorithm 2** given below, where the augmented Lagrangian is defined as $\mathcal{L}(x,z,d) = \|\widetilde{y} - (I_L \otimes D)x\|_2^2 + \lambda \mathrm{TV}(z) + \frac{\mu}{2}\|x - z - d\|_2^2$ with $\mu > 0$ being the penalty parameter, $q$ is the iteration number, $d \in \mathbb{R}^{ML}$ is the scaled dual variable, and the matrix $P \triangleq (I_L \otimes D)$. Note that Line 3 in **Algorithm 2** is nothing but the TV denoising operator with $(x^q - d^q)$ being the noisy input image. So, the denoising operator of Line 3 can be implemented by using the split Bregman method, whose implementation is freely available online[16]. Thus, the derivation of the CO-TV hyperspectral imaging algorithm has been completed.

**Algorithm 2** The CO-TV Hyperspectral Imaging Algorithm.

1: **Given** $\lambda := 0.1$, $\mu := 10$. Initialize $\boldsymbol{x}^0 := \boldsymbol{0}$, $\boldsymbol{d}^0 := \boldsymbol{0}$, and $q := 0$.
2: **repeat**
3: Update $\boldsymbol{z}^{q+1} \triangleq \arg\min_{\boldsymbol{z}} \ \mathcal{L}(\boldsymbol{x}^q, \boldsymbol{z}, \boldsymbol{d}^q) = \frac{1}{2}\|(\boldsymbol{x}^q - \boldsymbol{d}^q) - \boldsymbol{z}\|_2^2 + \frac{\lambda}{\mu}\mathrm{TV}(\boldsymbol{z})$;
4: Update $\boldsymbol{x}^{q+1} \triangleq \arg\min_{\boldsymbol{x}} \ \mathcal{L}(\boldsymbol{x}, \boldsymbol{z}^{q+1}, \boldsymbol{d}^q) := (2\boldsymbol{P}^T\boldsymbol{P} + \mu \boldsymbol{I}_L)^{-1}(2\boldsymbol{P}^T\widetilde{\boldsymbol{y}} + \mu(\boldsymbol{z}^{q+1} + \boldsymbol{d}^q))$;
5: Update $\boldsymbol{d}^{q+1} := \boldsymbol{d}^q + \boldsymbol{z}^{q+1} - \boldsymbol{x}^{q+1}$;
6: $q := q + 1$;
7: **until** the predefined stopping criterion is met.
8: Compute $\boldsymbol{x}^{\star} := \boldsymbol{x}^q$ and $\boldsymbol{X}^{\star} := \mathrm{vec}^{-1}_{M\times L}(\boldsymbol{x}^{\star})$.
9: **Output** the imaging result $\boldsymbol{X}^{\star}$.

**Supplementary Note 6: Definition of root-mean-square error (RMSE) and spectral angle mapper (SAM)**

The RMSE is described as:

$$\mathrm{RMSE} = \sqrt{\sum_{x=1}^{W}\sum_{y=1}^{H}\sum_{\mathrm{band}=1}^{N_{\mathrm{bands}}}\frac{\left[I_{\mathrm{image}}(x,y,\mathrm{band})-I_{\mathrm{GT}}(x,y,\mathrm{band})\right]^{2}}{N_{\mathrm{bands}}\times W\times H}} \tag{S10}$$

where $W$ and $H$ are the total pixels along $x$-direction and $y$-direction, respectively. $I_{\mathrm{image}}$ and $I_{\mathrm{GT}}$ are the intensity on the reconstructed hyperspectral images and ground truth, respectively. $N_{\mathrm{bands}}$ indicates the total number of wavelength bands.

The SAM is described as:

$$\mathrm{SAM} = \sum_{x=1}^{W}\sum_{y=1}^{H}\frac{\theta(x,y)}{W\times H} \tag{S11}$$

where $W$ and $H$ are the total pixels along $x$-direction and $y$-direction, respectively.

$$\theta = \cos^{-1}\left(\frac{v_{\mathrm{image}}\cdot v_{\mathrm{GT}}}{|v_{\mathrm{image}}|\,|v_{\mathrm{GT}}|}\right) \tag{S12}$$

$$v_{\mathrm{image}} = \left[I_{\mathrm{image}}^{\mathrm{band}\ 1}\quad I_{\mathrm{image}}^{\mathrm{band}\ 2}\cdots I_{\mathrm{image}}^{\mathrm{band}\ n}\right]^{T};\ v_{\mathrm{GT}} = \left[I_{\mathrm{GT}}^{\mathrm{band}\ 1}\quad I_{\mathrm{GT}}^{\mathrm{band}\ 2}\cdots I_{\mathrm{GT}}^{\mathrm{band}\ n}\right]^{T} \tag{S13}$$

**Supplementary Note 7: Discussion of the possibility for increasing the number of wavelength bands and the spectral range**

To achieve multiple resonant peaks in the optical spectrum without interleaving multiple meta-atoms or metasurfaces, it is essential to consider the design of the DBR substrate, the eigenmodes present in the nanostructure, and the coupling between the plasmonic nanostructure and the DBR substrate. The spectral range in which the multi-wavelength meta-atom can exhibit resonant behavior is primarily determined by the bandwidth of the reflection window of the DBR substrate. A wider reflection window allows for a broader working spectral range, enabling the meta-atom/MOFM to operate over a greater range of wavelengths.

The number of wavelength bands in the system is influenced by two key factors: the number of eigenmodes supported by the nanostructure and the cavity-like coupling between the nanostructure and the DBR substrate. Increasing the number of eigenmodes can be achieved by employing freeform nanostructures. Freeform nanostructures[17, 18], characterized by their anisotropic shape, adhere to the geometric phase method for phase modulation and enable a higher number of eigenmodes to be supported. Additionally, the number of wavelength bands can be extended by tuning the cavity-like coupling condition. Supplementary Figure 16a shows the simulation results of the LCP-to-RCP reflection spectrum for different thicknesses of the dielectric spacer $SiO_2$. In comparison to the original design with a 135-nm-thick $SiO_2$ spacer, which produced 4 peak wavelengths ranging from 480 nm to 650 nm (refer to Supplementary Figure 11), the number of wavelength bands increased to 8 and 12 with the 2000-nm-thick and 5000-nm-thick $SiO_2$ spacers, respectively. Notably, the simulations revealed the emergence of multi-resonant peaks in the blue spectral region below 480 nm, effectively filling the previously unexplored wavelength bands in the original design. Supplementary Figure 16b presents the simulated phase shift as a function of the structural rotation angle. It is evident from the plot that all the newly generated peak wavelengths align with the expected geometric phase profile. This observation confirms the effectiveness of the proposed method in increasing the number of wavelength channels, which reinforces the potential of the metasurface approach in expanding the spectral range and enhancing the versatility of multispectral/hyperspectral imaging systems.

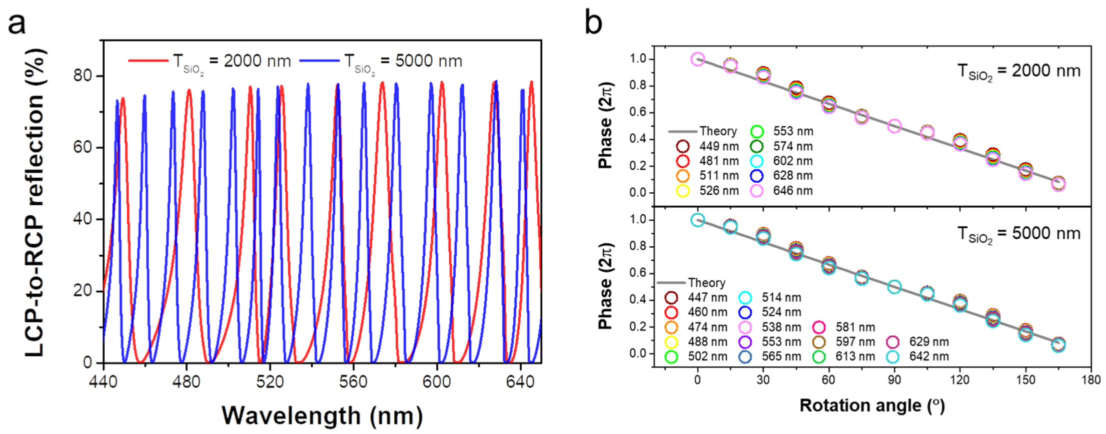


**Supplementary Figure 16. Simulated results of the designed meta-atom with 2000-nm-thick and 5000-nm-thick $SiO_2$ spacers. a** Numerical reflection spectrum of the designed meta-atom with different thicknesses of $SiO_2$. The DBR substrate and physical sizes of the Al nano-rod are the same as those listed in the main article. **b** The circularly cross-polarized phase spectrum as a function of structural rotation angle. All resonant peaks satisfy the geometric phase condition.

## Supplementary References


1. Chen, B. H., Wu, P. C., Su, V.-C., Lai, Y.-C., Chu, C. H., Lee, I. C., *et al.* GaN metalens for pixel-level full-color routing at visible light. *Nano Lett.* **17**, 6345-6352 (2017).
2. Lin, C. H., Ma, F., Chi, C. Y., Hsieh, C. H. A convex optimization-based coupled nonnegative matrix factorization algorithm for hyperspectral and multispectral data fusion. *IEEE Trans. Geosci. Remote Sens.* **56**, 1652-1667 (2018).
3. Lin, C. H., Lin, Y. C., Tang, P. W. ADMM-ADAM: A new inverse imaging framework blending the advantages of convex optimization and deep learning. *IEEE Trans. Geosci. Remote Sens.* **60**, 1-16 (2022).
4. Lin, C. H., Bioucas-Dias, J. M. An explicit and scene-adapted definition of convex self-similarity prior with application to unsupervised Sentinel-2 super-resolution. *IEEE Trans. Geosci. Remote Sens.* **58**, 3352-3365 (2020).
5. Chi, C.-Y. *et al.*, *Convex Optimization for Signal Processing and Communications: From Fundamentals to Applications*. CRC Press, Boca Raton, FL, 2017.
6. Zamir, S. W., Salman, Arora, A., Khan, S., Hayat, M., Khan, F. S., & Yang, M.-H. Restormer: Efficient transformer for high-resolution image restoration. *IEEE/CVF Conference on Computer Vision and Pattern Recognition (CVPR)*; 2022 p. 5728–5739.
7. Hu, X., Cai, Y., Lin, J., Wang, H., Yuan, X., Zhang, Y., Timofte, R., & Gool, V. L. HDNet: High-resolution dual-domain learning for spectral compressive imaging. *IEEE/CVF Conference on Computer Vision and Pattern Recognition (CVPR)*; 2022. p. 17542–17551.
8. Bioucas-Dias, J. M., Nascimento, J. M. P. Hyperspectral subspace identification. *IEEE Trans. Geosci. Remote Sens.* **46**, 2435-2445 (2008).
9. Boyd, S., Parikh, N., Chu, E., Peleato, B., Eckstein, J. Distributed optimization and statistical learning via the alternating direction method of multipliers. *Found. Trends Mach. Learn.* **3**, 1-122 (2011).
10. Fu, X., Jia, S., Zhuang, L., Xu, M., Zhou, J., Li, Q. Hyperspectral anomaly detection via deep plug-and-play denoising CNN regularization. *IEEE Trans. Geosci. Remote Sens.* **59**, 9553-9568 (2021).
11. Dian, R., Li, S., Kang, X. Regularizing Hyperspectral and multispectral image fusion by CNN denoiser. *IEEE Trans. Neural Netw. Learn. Syst.* **32**, 1124-1135 (2021).
12. Lin, T. H., Lin, C. H. Hyperspectral change detection using semi-supervised graph neural network and convex deep learning. *IEEE Trans. Geosci. Remote Sens.* **61**, 1-18 (2023).
13. Cai, Y., Lin, J., Lin, Z., Wang, H., Zhang, Y., Pfister, H., *et al.* MST++: Multi-stage spectral-wise transformer for efficient spectral reconstruction. *2022 IEEE/CVF Conference on Computer Vision and Pattern Recognition Workshops (CVPRW)*; 2022. pp. 744-754.
14. Zhao, Y., Po, L.-M., Yan, Q., Liu, W., Lin, T. Hierarchical regression network

for spectral reconstruction from rgb images. *2020 IEEE/CVF Conference on Computer Vision and Pattern Recognition Workshops (CVPRW)*; 2020. pp. 422-423.

15. Osher, S., Burger, M., Goldfarb, D., Xu, J., Yin, W. An iterative regularization method for total variation-based image restoration. *Multiscale Model. Simul.* **4**, 460-489 (2005).
16. Benjamin Tremoulheac (2023). Split Bregman method for total variation denoising (https://www.mathworks.com/matlabcentral/fileexchange/36278-split-bregman-method-for-total-variation-denoising), MATLAB Central File Exchange. Retrieved July 9, 2023.
17. Yang, J., Cui, K., Cai, X., Xiong, J., Zhu, H., Rao, S., *et al.* Ultraspectral imaging based on metasurfaces with freeform shaped meta-atoms. *Laser Photon. Rev.* **16**, 2100663 (2022).
18. Zou, X., Zhang, Y., Lin, R., Gong, G., Wang, S., Zhu, S., *et al.* Pixel-level Bayer-type colour router based on metasurfaces. *Nat. Commun.* **13**, 3288 (2022).
